# Preconditioning influences mesenchymal stem cell properties *in vitro* and *in vivo*

**DOI:** 10.1111/jcmm.13492

**Published:** 2018-02-01

**Authors:** Chenxia Hu, Lanjuan Li

**Affiliations:** ^1^ Collaborative Innovation Center for Diagnosis and Treatment of Infectious Diseases State Key Laboratory for Diagnosis and Treatment of Infectious Diseases School of Medicine First Affiliated Hospital Zhejiang University Hangzhou Zhejiang China

**Keywords:** preconditioning, mesenchymal stem cell, stemness, *in vitro*, *in vivo*

## Abstract

Various diseases and toxic factors easily impair cellular and organic functions in mammals. Organ transplantation is used to rescue organ function, but is limited by scarce resources. Mesenchymal stem cell (MSC)‐based therapy carries promising potential in regenerative medicine because of the self‐renewal and multilineage potency of MSCs; however, MSCs may lose biological functions after isolation and cultivation for a long time *in vitro*. Moreover, after they are injected *in vivo* and migrate into the damaged tissues or organs, they encounter a harsh environment coupled with death signals due to the inadequate tensegrity structure between the cells and matrix. Preconditioning, genetic modification and optimization of MSC culture conditions are key strategies to improve MSC functions *in vitro* and *in vivo*, and all of these procedures will contribute to improving MSC transplantation efficacy in tissue engineering and regenerative medicine. Preconditioning with various physical, chemical and biological factors is possible to preserve the stemness of MSCs for further application in studies and clinical tests. In this review, we mainly focus on preconditioning and the corresponding mechanisms for improving MSC activities *in vitro* and *in vivo*; we provide a glimpse into the promotion of MSC‐based cell therapy development for regenerative medicine. As a promising consequence, MSC transplantation can be applied for the treatment of some terminal diseases and can prolong the survival time of patients in the near future.

## Introduction

Various diseases and toxic factors easily impair cellular and organic functions in mammals; parenchymal cells and stromal cells move into an apoptotic state, accelerating the development of injury or disease. If the functions cannot be repaired in a timely manner, multiple complications will occur as a consequence. Previously, organ transplantation was the only effective repair for tissue injury, whereas cell transplantation has gradually become a burgeoning route for regenerative medicine. However, transplantation of primary cells is limited by scarce resources; therefore, stem cell transplantation becomes a hot topic in current tissue engineering. In general, stem cells are classified as embryonic stem cells (ESCs), induced pluripotent stem cells (iPSCs) and adult stem cells such as mesenchymal stem cells (MSCs) and other tissue‐specific stem cells 1. ESCs are stem cells isolated from the inner cell mass of early‐stage embryos; they can proliferate indefinitely without a senescent phenotype and differentiate into all cell lineages 2. Transcription factors such as OCT4 and NANOG are indispensable for maintaining the high pluripotency state of ESCs 3, and then, iPSCs were generated via conversion of somatic cells into an ESC‐like pluripotent state by overexpression of several vital transcription factors 4. Although both of them have extreme pluripotency, unlimited self‐renewal and multilineage potency, their application should be considered carefully because of potential ethical problems, safety issues and low efficiency 5. MSCs, which are prevalent in tissues, exist in almost all kinds of tissues, and they are adult stem cells with multilineage potentials 6. There are no ethical issues associated with their use, and they serve as a promising cell source for cell‐based therapy to maintain homeostasis under physiological and pathological conditions 7, 8. Furthermore, the low immunological rejection rate permits their autotransplantation and allogeneic transplantation applications 9. MSCs exhibit plastic adherence and fibroblast spindle‐like‐shaped morphology when cultured *in vitro* and can highly proliferate and differentiate towards adipocytes, osteocytes, chondrocytes, hepatocytes, endothelial cells, cardiomyocytes, neural cells, etc. 1, 10, 11, 12. MSCs have been reported to exert various effects on host cells or organs via immunomodulation 13: pro‐angiogenic 14, antiapoptotic 15 and antioxidative effects 16 and activation of local quiescent stem cells 17. In fact, MSCs together with local somatic cells establish cell‐to‐cell interactions and produce autocrine and paracrine factors 18, and culture medium isolated from MSCs also contains biological factors including mRNAs, microRNAs and enzymes that protect cells or organs from further damage 19. Moreover, cell fusion of MSCs also contributes to the repair of tissues or organ function 13, 14; it occurs rarely and is classified into two types, namely homotypic and heterotypic cell fusions, with the former occurring between the same lineage, while the latter occurs between different lineages 15. In addition, evidence demonstrated that MSCs could still transdifferentiate into cardiomyocytes or neural cells at low rates after infusion or injection in mammals although MSCs mainly promoted the regeneration of injured organs through paracrine mechanism 12, 16.

**Figure 1:**
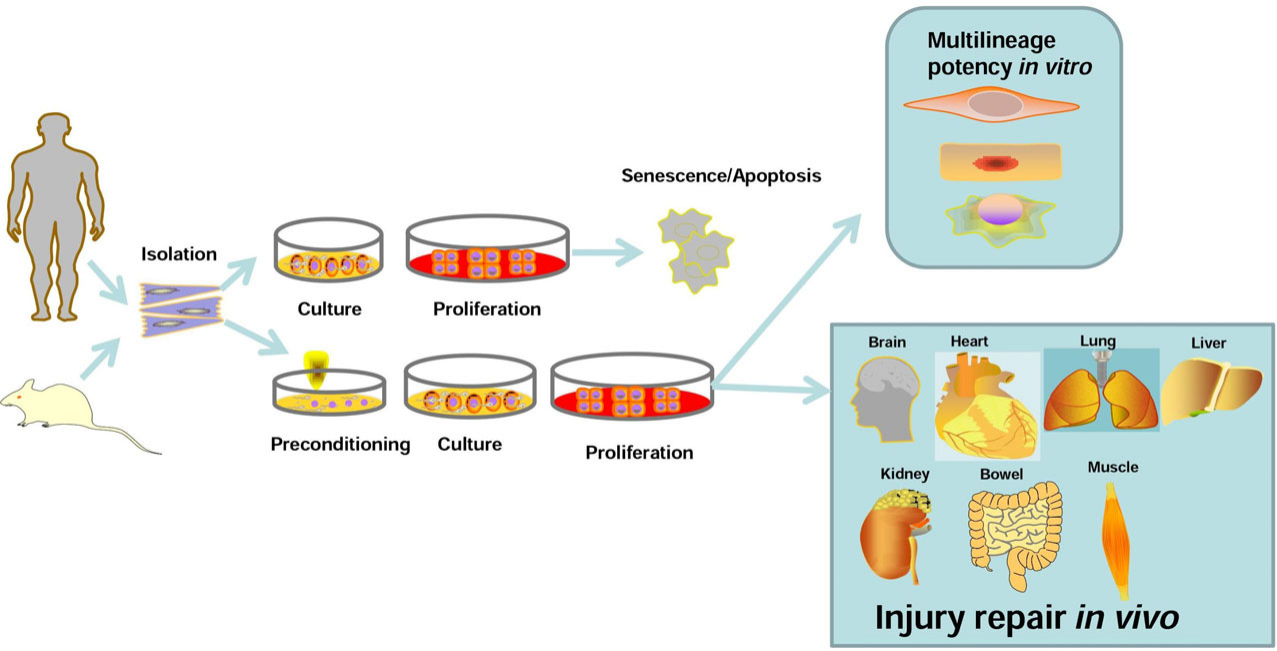
After isolation *in vitro*, MSCs can be pretreated with various protective factors to improve survival rate and further enhance its application *in vitro* and *in vivo*.

MSCs reside in the general microenvironment with low oxygen tension (i.e. 1–5% O_2_) *in vivo*, while the oxygen concentration of the general culture environment (i.e. 20–21% O_2_) is higher than their original living environment 17, 20. The changed culture oxygen tension *in vitro* may decrease the cell activities, including proliferation, differentiation, the anti‐inflammatory response, and also decrease the cell activities for repairing dysfunctional organs 21, 22. MSCs are generally deprived of nutrients and oxygen after isolation *ex vivo*, and the addition of trophic factors is not enough to maintain the cell activities for further application 20. MSCs isolated from unhealthy individuals or those that have proliferated for a long period will have an impaired self‐renewal ability and physiological state 23, 24. In contrast, transplanted MSCs are confronted with apoptosis or senescence attributed to harsh environmental conditions, anoikis and inflammation induced by damaged tissues or organs 25, which leads to an imbalance between the generation of reactive oxygen species (ROS) and antioxidant mechanisms 26. Inflammatory cells, such as neutrophils, monocytes and macrophages, which are recruited by chemokines or cytokines, will induce apoptosis and inactivate the cytoprotective production of nitric oxide (NO) 27. The acute inflammatory response promotes angiogenesis and the recruitment of progenitor cells, while chronic inflammation inhibits the recruitment and survival of local progenitor cells and implanted MSCs 28. To this end, MSCs have been shown to protect tissues and organs from external injury; however, investigating more effective routes to improve their therapeutic effects is still necessary (Fig. 1).

**Figure 2:**
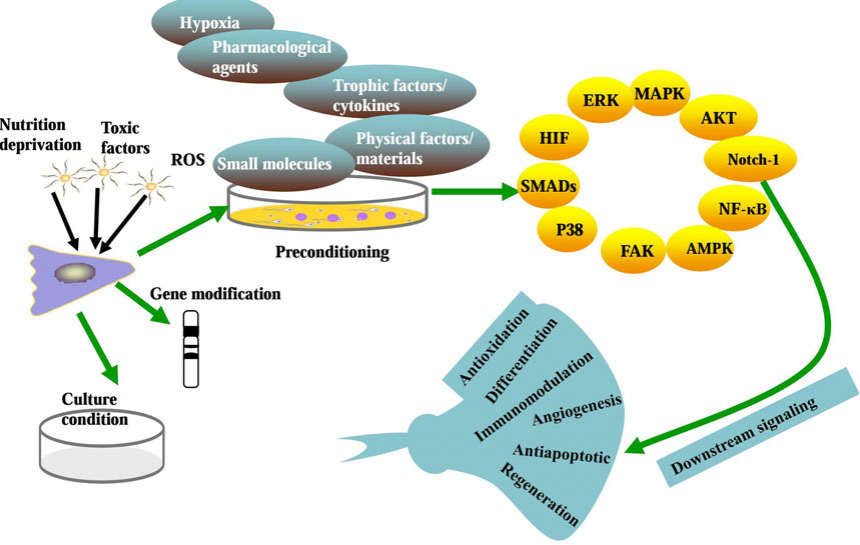
Preconditioning, genetic modification and optimization of MSC culture conditions are key strategies to improve MSC function *in vitro* and *in vivo*, and preconditioning is effective at activating various important signalling pathways for protecting cells and organs from injury.

Preconditioning, genetic modification and optimization of MSC culture conditions are key strategies to improve MSC function *in vitro* and *in vivo*. All of these procedures contribute to improving MSC transplantation efficacy in tissue engineering and regenerative medicine 29. Recently, studies have demonstrated that pretreated MSCs showed better cell survival, increased differentiation efficacy, enhanced paracrine effects and an improved homing ability into injury sites 29, 30, 31. Preconditioning of MSCs by hypoxia, pharmacological agents, chemical agents, trophic factors, cytokines and physical factors prior to their application is capable of initiating survival signalling to counter the rigorous harsh microenvironment for MSC transplantation application (Fig. 2). However, the optimization of these external factors for MSC preconditioning and the underlying mechanisms should be further investigated to improve MSC therapeutic effects. Currently, the mechanisms of preconditioning with hypoxia and other important strategies (Tables 1 and 2) have been widely investigated in experimental studies and clinical trials. In this review, we mainly focus on the preconditioning and the corresponding mechanisms for improving MSC activities *in vitro* and *in vivo*. In this way, we provide a glimpse in the promotion of MSC‐based cell therapy development for regenerative medicine. As a promising consequence, MSC transplantation can be applied for the treatment of some terminal diseases and can prolong the survival time of patients in the near future.

**Table 1:** Hypoxia and the potential mechanisms for preconditioning on MSCs

O_2_ content	Effect	Mechanism	Reference
0.5%	Counteracts the deficiency of adipose‐derived MSCs from older donors and highly improves their differentiation capacity	Acts as a protective factor	23
1%	Prevents the apoptosis of MSCs	Increases the secretion of angiogenic factors, VEGF and basic fibroblast growth factor (BFGF) in MSCs	36
1%	Decreases the sensitivity of MSCs to the ischaemic microenvironment without changing their biological behaviour, immunophenotype or karyotype	Increases the metabolic activity and decreases the caspase‐3/7 activity and lactate dehydrogenase release of MSCs	37
2%	Decreases tumorigenic potential of MSCs	Down‐regulates the expression levels of tumour‐suppressor genes and TERT and the suboptimal double‐stranded DNA breaks in MSCs	17
3%	Improves the genetic stability and chromosome stability and guarantees the safety of MSCs	Decreases the incidence of aneuploidy in MSCs	38
5%	Enhances the clonogenic potential and proliferation rate of MSCs	Up‐regulates the vascular endothelial growth factor (VEGF) secretion in MSCs	35
5%	Exerts no effect on the phenotype or differentiation ability of MSCs but significantly enhances the autophagy progress	Increases the expression of HIF‐1α and activates the AMPK/mTOR signalling pathway	39
0.1–0.3%	Promotes neurogenesis and neurological functional recovery	Promotes the secretion of various growth factors including brain‐derived neurotrophic factor (BDNF), glial cell line‐derived neurotrophic factor (GDNF) and VEGF	40
0.5%	Improves the motor and cognitive function of the animal models	Promotes the secretion of growth factors including hepatocyte growth factor (HGF) and VEGF	42
0.5%	Suppresses microglia activity in the brain and promotes locomotion recovery	Up‐regulates the expression levels of HIF‐1α, the VEGF receptor, erythropoietin (EPO), the EPO receptor, stromal‐derived factor‐1 (SDF‐1) and CXC chemokine receptor 4 (CXCR4) but decreases the release of pro‐inflammatory cytokines	43
1%	Promotes liver regeneration in massive hepatectomy models	Increases the expression of cyclin D1 and VEGF, enhances the proliferation of hepatocytes and increases the liver weight/bodyweight ratio	41
1%	Improves the intracavernosal pressure and erectile function in diabetes models	Up‐regulates the release of angiogenesis‐ and neuroprotection‐related factors including VEGF, the VEGF receptor, angiotensin, BFGF, BDNF, GDNF, SDF‐1 and CXCR4; up‐regulates the expression levels of NO synthases, endothelial markers and smooth muscle markers	45
1.5%	Compensates the loss of lung functions in idiopathic pulmonary fibrosis models	Improves the proliferation, migration, angiogenesis, antioxidant, antiapoptotic and antifibrotic properties of implanted MSCs	46
1.5%	Guarantees the safety of cell transplantation	Inhibits the malignant transformation of MSCs	47
1–7%	Repairs the injury in a murine hindlimb ischaemia model	Activates the HIF‐1α/GRP78/Akt signal axis	48
2%	Promotes the recovery of the ischaemic tissue	Improves the expression of prion protein (PrPC), activates PrPC‐dependent JAK2 and STAT3 signalling pathways, and then up‐regulates the activity of superoxide dismutase and catalase	49
2%	Inhibits the rabbit femoral head osteonecrosis	Increases the angiogenesis function and decreases the tissue apoptosis	50
5%	Facilitates revascularization in diabetic lower limb ischaemia (DLLI)	Increases the expression levels of angiogenin, matrix metallopeptidase (MMP)‐9, VEGF‐1α and HIF‐1α and activates the p‐AKT signalling pathway	51

**Table 2:** Other important agents and the potential mechanisms for preconditioning on MSCs

Type	Agent	Effect	Mechanism	Reference
Clinical drugs	Lenalidomide	Up‐regulates the erythroid and myeloid colony formation of early haematopoietic progenitors of MSCs	Reduces the release of stromal‐derived factor 1‐alpha and cobblestone area formation	53
	Vitamin E	Reduces oxidative stress and senescence induced by hydrogen peroxide (H_2_O_2_) in MSCs	Up‐regulates the expression levels of proliferative markers and transforming growth factor‐beta (TGF‐β), while it reduces the expression levels of apoptosis‐related genes and the release of VEGF and lactate dehydrogenase (LDH)	54
	Low‐dose LPS	Inhibits the apoptosis of MSCs induced by hypoxia and serum deprivation (H/SD)	Preserves the mitochondrial membrane potential and inhibits cyto C release; activates the ERK signalling pathway	55
	Sevoflurane	Maintains the survival and migration rates of MSCs under condition of H/SD	Up‐regulates the expression levels of HIF‐1α, HIF‐2α, VEGF and p‐Akt/Akt	56
	Valproic acid	Promotes hepatogenic differentiation of MSCs	Activates the AKT and ERK signalling pathways	57
	Astragaloside IV	Promotes the proliferation of MSCs and inhibits the high glucose‐induced expression of Toll‐like receptor 4 (TLR4) in MSCs	Decreases the translocation of NF‐κB p65 and increases the level of MMP‐2	58
	Apple extract	Enhances the proliferative ability of MSCs	Up‐regulates the phosphorylation of p44/42 MAPK, mTOR, p70S6K, S6RP, eIF4B and eIF4E and up‐regulates the release of VEGF and interleukin‐6	59
	Icariside II	Promotes the proliferation and osteogenic differentiation of MSCs	Up‐regulates the PI3K/AKT/mTOR/S6K1 signalling pathways	60
	Genistein	Enhances cell proliferation and adipogenic differentiation of MSCs but inhibits the osteogenic progress	Up‐regulates the expression of PPARγ	61
	Oxytocin	Promotes the cell proliferation and migration of MSCs; protects against the H/SD‐induced cytotoxic and apoptotic effects on MSCs	Activates the Akt/ERK1/2 pathway	62
	Deferoxamine	Increases the migration of MSCs *in vitro* and homing ability of MSCs *in vivo*	Increases the expression of HIF‐1α, CXCR4, C‐C motif chemokine receptor 2 (CCR2), MMP‐2 and MMP‐9	63
	Atorvastatin	Enhances the cardiac function and improves post‐implantation survival of MSCs	Activates the eNOS/NO system	24
	2,4‐Dinitrophenol (DNP)	Improves cardiac function and reduces scar formation after MSC transplantation	Up‐regulates angiogenesis in myocardial infarction models	64
	Angiotensin II	Decreases the degree of cardiac fibrosis and infarct size after MSC transplantation	Up‐regulates the release of growth factors	65
	Angiotensin receptor blockers (ARBs)	Maintains the left ventricular ejection fraction after MSC transplantation	Improves the cardiomyogenic transdifferentiation of MSCs	66
	OT	Improves cardiac function and reduces fibrosis in a rat myocardial infarction model after MSC transplantation	Up‐regulates the expression level of Krüppel‐like factor 2 (KLF2) and angiogenic differentiation ability of MSCs	67
	Melatonin	Protects brain function from injury	Activates the ERK signalling pathway	69
	Rapamycin (RAP)	Promotes the osteogenesis of MSCs *in vitro* and *in vivo*	Activates the autophagy	70
	Vitamin E	Impedes the progression of osteoarthritis and increases potential to treat osteoarthritis after MSC transplantation	Increases the proteoglycan content in the cartilage matrix	54
	All‐trans retinoic acid (ATRA)	Enhances the tube formation and the *in vivo* wound healing ability after MSC transplantation	Increases the expression levels of cytochrome c oxidase (COX)‐2, HIF‐1, CXCR4, CCR2, VEGF, angiogenin‐2 and angiogenin‐4 in MSCs	71
	Polyribocytidylic acid	Rescues the trinitrobenzene sulphonate (TNBS)‐induced colitis mouse models after MSC transplantation	Activates the Notch‐1 signalling pathway	72
Small molecules	BAY 11‐708	Blocks the pro‐angiogenesis and antiapoptosis function of MSCs	Inhibits the NF‐κB activity	73
	LL‐37	Enhances the MSC proliferation and migration	Activate the MAPK signalling pathway	74
	Dimethyloxalylglycine (DMOG)	Improves the therapeutic effects of MSCs for reducing heart infarct size and promoting functional repair in myocardial infarction	Increases the expression levels of survival and angiogenic factors including HIF‐1α, VEGF, glucose transporter 1 and phospho‐AKT in MSCs	76
	JI‐34	Enhances the differentiation into endothelial tube cells *in vitro* and improves the engraftment of MSCs into hemic hindlimb muscles for repairing injured parts	Serves as a growth hormone‐releasing hormone agonist	78
Cytokines	Stromal‐derived factor‐1 (SDF‐1)	Protects MSCs from H_2_O_2_‐induced apoptosis	Enhances the proliferation, migration, and survival rate of MSCs; up‐regulates the release of angiogenic cytokines and activates the AKT and ERK signalling pathways	81
	TGF‐β1	Drives MSC fate towards osteoblasts generation *in vitro*, while it inhibits adipogenic differentiation of MSCs; switches MSCs from adipogenesis into osteogenesis	Activates SMAD/C/EBPs/PPARγ signalling pathways	82
	TNF‐α	Enhances proliferation, mobilization and osteogenic differentiation of MSCs	Activates the ERK1/2 and MAPK signalling pathways	83
	Interferon (IFN)‐γ	Suppresses natural killer (NK) activation and NK‐mediated cytotoxicity partly	Up‐regulates the synthesis of indoleamine 2,3‐dioxygenase (IDO) and prostaglandin E2	84
	IL‐1 and TNF‐α	Inhibits the osteogenesis and adipocyte generation of MSCs	Activates the canonical NF‐κB signalling, IL‐1R1/MyD88 signalling pathway	85, 86
	TNF‐α and IFN‐γ	Promotes the generation of anti‐inflammatory M2 macrophages and suppresses the human peripheral blood mononuclear cell proliferation	Increases the production of IDO	87
	IL‐1β	Improves the migration ability of MSCs	Increases the expression levels of various cytokines and chemokines, as well as adhesion molecules in MSCs	89
	TGF‐β1	Induces MSC migration into the remodelling sites and couples bone formation and resorption	Activates the canonical SMADs signalling pathway or the non‐canonical signalling pathways involving AKT, ERK1/2, FAK and p38	91
	TGF‐β1	Prolongs the effective therapy time in damaged lungs	Improves the survival of MSCs and enhances the expression of extracellular matrix components, particularly fibronectin in damaged lungs	92
	Oncostatin M (OSM)	Maintains pulmonary respiratory function, and down‐regulates the release of inflammatory and fibrotic factors in bleomycin‐induced lung fibrotic mice	Increases the expression of type 2 OSM receptor and HGF in MSCs	93
	IFN‐α	Improves the therapeutic effects on dextran sodium sulphate (DSS)‐ and TNBS‐induced colitis after MSC transplantation	Increases the migration potential of MSCs and inhibits Th1 inflammatory responses	94
	Migration inhibitory factor (MIF)	Maintains the proliferation and survival of aged MSCs *in vivo*	Improves the secretion of various growth factors including VEGF, BFGF, HGF and insulin‐like growth factor (IGF)	95
Physical factors	Low‐level lasers	Improves the proliferation rate, enhances mitochondrial biogenesis and improves the migration ability of MSCs	Up‐regulates the generation of ROS and NO, activates the ERK1/2 and FAK signalling pathways and up‐regulates the expression levels of HGF and platelet‐derived growth factor (PDGF)	96
	Pulsed electromagnetic fields (PEMF)	Decreases the cell death rate of MSCs	Up‐regulates the expression of AKT and RAS	97
	Silica	Increases the proliferation of MSCs or induces a slight increase in apoptosis	Improves the phosphorylation of ERK1/2; up‐regulates the phosphorylation of p38	98
	Mechanical stretch	Increases the angiogenic capacity and survival rate of MSCs	Up‐regulates the activities of VEGFA and NF‐κB p65	73
	GFc7	Increases the quality and differentiation rate of MSCs	Improves cell proliferation, expression of pluripotency genes and homing markers and antioxidative defence; represses the spontaneous differentiation‐related genes while it enhances the levels of specific differentiation‐related genes	99
	Selenite	Recovers the osteoblastic differentiation of MSCs	Inhibits the activation of the ERK signalling pathway	100
	Carboxyl‐terminated hyperbranched polyester (CHBP)	Provides more resistance for MSCs to starvation‐induced oxidative stress in a dose‐dependent manner	Maintains the MMP and mitochondrial membrane integrity and activates the Nrf2/Sirt3/FoxO3a pathway	101

## Hypoxia preconditioning for improving the cell activities of MSCs

O_2_ is essential for cellular homeostasis as the lack of O_2_ is involved in the pathogenesis of some diseases with high morbidity and mortality 32. Hypoxia preconditioning including culturing for 15 min at 2.5% O_2_, reoxygenation for 30 min at 21% O_2_ and hypoxia preconditioning for 72 hrs at 2.5% O_2_ significantly improves the proliferation and migration abilities of MSCs *in vitro* 33. Hypoxia reduces the cell viability and proliferation of MSCs but the following reoxygenation promotes the recovery of MSCs, and the process of hypoxia and reoxygenation (H/R) improves the expression of pro‐survival genes and various trophic factors in MSCs 34; moreover, hypoxia or H/R has been proven to promote multipotency of MSCs in determined microenvironments *in vitro* 33, 35. Considering the advantages of hypoxia or H/R, current studies have focused on the optimization of oxygen concentrations to improve the cell activities and therapeutic effects of MSCs. Hypoxia at 0.5% O_2_ for 24 hrs can act as a protective factor to effectively counteract the deficiency of adipose‐derived MSCs from older donors and highly improve their differentiation capacity 23. When incubated in low‐serum medium, MSCs will move into an apoptotic state, but hypoxia preconditioning (1% O_2_) can prohibit the damage via increasing the secretion of angiogenic factors, VEGF and basic fibroblast growth factor (BFGF) 36. Moreover, 1% O_2_ also increases the metabolic activity and decreases the caspase‐3/7 activity and lactate dehydrogenase release of MSCs, thus decreasing the sensitivity of MSCs to the ischaemic microenvironment without changing their biological behaviour, immunophenotype or karyotype 37. The decreased tumorigenic potential of MSCs induced by 2% O_2_ has been demonstrated to be mediated by the down‐regulation of the expression levels of tumour‐suppressor genes and TERT and the induction of suboptimal double‐stranded DNA breaks in MSCs *in vitro* 17. Intriguingly, a hypoxic condition at 3% O_2_ decreases the incidence of aneuploidy in MSCs compared to that in the normoxic condition at 20% O_2_, indicating that pretreatment with hypoxia improves the genetic stability and chromosome stability and guarantees the safety of MSCs *in vitro* 38. Culture in 5% ambient O_2_ consistently enhances the clonogenic potential and proliferation rate of MSCs via up‐regulation of vascular endothelial growth factor (VEGF) secretion 35. Liu *et al*. 39 demonstrated that 5% O_2_ exerted no effect on the phenotype or differentiation ability of MSCs but significantly enhanced the autophagy by increasing the expression of HIF‐1α and the activation of the AMPK/mTOR signalling pathway. While Boyette *et al*. 35 proved that 5% ambient O_2_ improved the osteogenesis and chondrogenesis of MSCs expanded under normoxia, while it inhibited the adipogenesis and chondrogenesis of MSCs expanded under three‐dimensional pellet conditions.

After transplantation *in vivo*, the pathological environment significantly decreases the self‐renewal and survival rate of MSCs. Evidence has shown that transplantation of hypoxia‐pretreated MSCs enhanced the therapeutic effects via up‐regulating the secretion of cytokines and growth factors 40, 41. After hypoxia preconditioning (0.1–0.3% O_2_), transplanted MSCs migrate into peri‐injury regions in the intracerebral haemorrhagic stroke brain and promote neurogenesis and neurological functional recovery through secreting various growth factors, including brain‐derived neurotrophic factor (BDNF), glial cell line‐derived neurotrophic factor (GDNF) and VEGF 40. In traumatic brain injury rats, hypoxia preconditioning at 0.5% O_2_ promotes MSCs to secrete more growth factors, including hepatocyte growth factor (HGF) and VEGF, to improve the motor and cognitive functions of the animal models 42. In addition to the above‐mentioned growth factors, the expression levels of HIF‐1α, the VEGF receptor, erythropoietin (EPO), the EPO receptor, stromal‐derived factor‐1 (SDF‐1) and CXC chemokine receptor 4 (CXCR4) were improved, while the release of pro‐inflammatory cytokines was decreased by MSCs preconditioned with hypoxia at 0.5% O_2_. Furthermore, these pretreated MSCs have been demonstrated to suppress microglia activity in the brain and promote locomotion recovery more effectively than a normoxia‐cultured MSC group 43. Although MSCs that are pretreated by hypoxia at 0.5% O_2_ can improve the left ventricular function of monkeys with myocardial infarction more effectively than normoxia‐cultured MSCs, they are not able to increase the occurrence of arrhythmogenic complications 44. MSCs that are pretreated under 1% O_2_ have been shown to promote liver regeneration in massive hepatectomy models via increasing expression of cyclin D1 and VEGF, enhancing proliferation of hepatocytes and increasing the liver weight/bodyweight ratio 41. Sublethal oxygen concentration (1%) up‐regulates the release of angiogenesis‐ and neuroprotection‐related factors including VEGF, the VEGF receptor, angiotensin, BFGF, BDNF, GDNF, SDF‐1 and CXCR4 in MSCs, and transplantation of these cells significantly up‐regulates the expression levels of NO synthases, endothelial markers and smooth muscle markers and also improves the intracavernosal pressure and erectile function in diabetes models 45. Preconditioning with 1.5% O_2_ enhances the activities of MSCs and effectively improves the proliferation, migration, angiogenesis, antioxidant, antiapoptotic and antifibrotic properties of implanted MSCs to compensate the loss of lung functions in idiopathic pulmonary fibrosis models 46. Most importantly, preconditioning with hypoxia at 1.5% O_2_ inhibits the malignant transformation of MSCs after transplantation and thus highly guarantees the safety of cell transplantation 47. After transplantation in a murine hindlimb ischaemia model, MSCs that are pretreated by 1% to 7% O_2_ activate the HIF‐1α/GRP78/Akt signal axis for repairing the injury 48; moreover, 2% O_2_ improves the expression of prion protein (PrPC), activates PrPC‐dependent JAK2 and STAT3 signalling pathways and then up‐regulates the activity of superoxide dismutase and catalase to inhibit the oxidative stress‐induced apoptosis of MSCs and promote the recovery of the ischaemic tissue 49. Transplantation of 2% O_2_‐treated MSCs significantly increases the angiogenesis function and decreases the tissue apoptosis in rabbit femoral head osteonecrosis compared to that observed in 20% O_2_‐treated MSCs 50. MSCs that are pretreated with hypoxia at 5% O_2_ can not only improve the migratory and tube‐forming capacities of endothelial cells *in vitro* but also facilitate revascularization in diabetic lower limb ischaemia (DLLI) via increasing the expression levels of angiogenin, matrix metallopeptidase (MMP)‐9, VEGF‐1α and HIF‐1α and activation of the p‐AKT signalling pathway 51. Although most studies have proven that hypoxia is a protective factor for MSCs *in vitro* and *in vivo*, determining the optimal oxygen concentration for improving the survival rate and therapeutic effects of MSCs is still necessary.

## Preconditioning with pharmacological or chemical agents for MSC‐based regenerative medicine

Various drugs exert therapeutic effects in different diseases and protect organs from further loss of function, thus elongating the long‐term survival rate of patients. In current studies, specialists focus on investigating the underlying mechanisms of drugs to protect MSCs from injury and improve their application in regenerative medicine. Although special medications have been demonstrated to exert protective effects on MSCs, as a premise, extremely high concentrations of drugs will impair the function of MSCs. Thus, exploring the optimal concentration of drugs for MSCs *in vitro* and *in vivo* is a prerequisite. High concentrations of zoledronic acid inhibited the proliferation and osteogenic differentiation of bone marrow‐derived MSCs, while low concentrations of zoledronic acid played the opposite role without influencing their immunomodulatory properties 52. Preconditioning with drugs is presumed to be responsible for protecting against ischaemic injury during stem cell transplantation and further activating endogenous cellular machinery for regeneration.

Under the pathological state in various diseases, the self‐renewal and differentiation abilities of MSCs are undoubtedly decreased, thus limiting the supply of cell resources for basic application. For instance, MSCs isolated from those with low‐ but not high‐risk myelodysplastic syndrome demonstrated a lower erythroid and myeloid colony formation of early haematopoietic progenitors; fortunately, preconditioning with lenalidomide effectively rescued the dysfunction in the disease‐derived MSCs 53. Because hydrogen peroxide (H_2_O_2_) induces oxidative stress and senescence in MSCs *in vitro*, vitamin E pretreatment counteracts the injury and up‐regulates the expression levels of proliferative markers and transforming growth factor‐beta (TGF‐β), while it reduces the expression levels of apoptosis‐related genes and the release of VEGF and lactate dehydrogenase (LDH) *in vitro* 54. Hypoxia and serum deprivation (H/SD) *in vitro* initiates apoptosis of MSCs, while preconditioning of MSCs with low‐dose lipopolysaccharide (LPS) preserves the mitochondrial membrane potential and inhibits cyto C release in H/SD‐cultured MSCs; LPS preconditioning also decreases the expression of connexin 43 via regulation of the ERK signalling pathway, thus stabilizing the cell membrane of MSCs 55. Preconditioning with sevoflurane up‐regulates the expression levels of HIF‐1α, HIF‐2α, VEGF and p‐Akt/Akt and prevents the initiation of apoptosis and loss of the mitochondrial membrane potential, thus maintaining the survival and migration rates of MSCs after H/SD 56. In addition, valproic acid (VPA), a histone deacetylase inhibitor with anticonvulsive function, takes part in the promotion of hepatogenic differentiation via activation of the AKT and ERK signalling pathways and up‐regulation of the expression of endodermal genes in MSCs *in vitro* 57. Chinese medicines and some natural extracts have also been applied to protect MSCs from injury and enhance the differentiation content of MSCs *in vitro*. Astragaloside IV, which can resist oxidative stress‐induced injury, has been used to promote the proliferation of MSCs and inhibit the high glucose‐induced expression of Toll‐like receptor 4 (TLR4) in MSCs via decreasing the translocation of NF‐κB p65 and increasing MMP‐2 expression 58. Apple extract dose dependently enhances the proliferative ability of adipose‐derived MSCs via phosphorylation of p44/42 MAPK, mTOR, p70S6K, S6RP, eIF4B and eIF4E and up‐regulation of the release of VEGF and interleukin‐6 59. Preconditioning with icariside II (ICSII) has been demonstrated to promote the proliferation and osteogenic differentiation of bone marrow‐derived MSCs via up‐regulating PI3K/AKT/mTOR/S6K1 signalling pathways, and wortmannin or rapamycin neutralizes the transition and acts as antagonists for this type of Chinese medicine 60. Genistein, which is a type of phytoestrogen, has anti‐inflammatory and antioxidative abilities; it exerts different effects on MSCs as it enhances cell proliferation and adipogenic differentiation of MSCs but inhibits the osteogenic progress by up‐regulating PPARγ expression 61. On the other hand, hormones that can maintain the homeostasis of mammals and protect organs from injury contribute to the preconditioning of MSCs *in vitro*. Oxytocin (OT), which is secreted from the hypothalamus, induces activation of the Akt/ERK1/2 pathway to promote the cell proliferation and migration of MSCs, thus protecting against the H/SD‐induced cytotoxic and apoptotic effects on MSCs 62.

Preconditioning of MSCs with various drugs *in vitro* also improves the therapeutic effects of MSCs *in vivo* for multiple diseases by repairing the lost functions via regulation of various pathways. Deferoxamine (DFO), a type of iron chelator, increases the migration *in vitro* and homing ability *in vivo* of MSCs via increasing the expression of HIF‐1α, CXCR4, C‐C motif chemokine receptor 2 (CCR2), MMP‐2 and MMP‐9 but can be significantly down‐regulated by an HIF‐1α inhibitor, namely 2‐methoxyestradiol 63. Atorvastatin is widely used in coronary heart disease for inhibiting hyperlipidaemia, preconditioning with which has been demonstrated to enhance cardiac function and improve post‐implantation survival via activation of the eNOS/NO system in MSCs 24. 2,4‐Dinitrophenol (DNP)‐pretreated MSCs significantly improve cardiac function and reduce scar formation via up‐regulation of angiogenesis in myocardial infarction models 64. The renin–angiotensin–aldosterone system (RAAS) takes part in physiological processes by responding to external stimuli, and pretreatment with the derivatives of RAAS can effectively influence MSC activities *in vivo*. Transplantation of angiotensin II‐pretreated MSCs leads to less cardiac fibrosis, smaller infarct size via up‐regulation and the release of growth factors in the myocardial infarcted myocardium, whereas there is no promotion of cardiogenic differentiation in MSCs 65. Pretreatment with angiotensin receptor blockers (ARBs) increases the cardiomyogenic differentiation efficiency of MSCs *in vitro*. Furthermore, transplantation of ARB‐pretreated MSCs can significantly maintain the left ventricular ejection fraction *in vivo* 66. Streptozotocin is widely used to produce diabetes models, and MSCs isolated from these models show severely impaired proliferation and angiogenic activities. OT significantly reversed the self‐renewal and differentiation ability of these MSCs *in vivo*. Consequently, transplantation of OT‐pretreated diabetes‐derived MSCs significantly improves cardiac function and reduces fibrosis in a rat myocardial infarction model 67. VPA pretreatment significantly up‐regulates the expression levels of neuroprogenitor markers including nestin, Musashi, prominin 1 and glial fibrillary acidic protein and consequently increases the number and neurite length of neurogenic MSCs 68. In a cerebral ischaemia model, melatonin pretreatment increases the post‐transplantation survival of implanted MSCs and protects brain function from injury via activating the ERK signalling pathway 69. In spite of cardiogenic differentiation and neural differentiation, other drugs have been used to improve the other differentiation abilities of MSCs *in vivo*. Rapamycin (RAP), which is an antifungal antibiotic, can significantly promote the osteogenesis of MSCs *in vivo* by the activation of autophagy, but the effect can be reversed by an autophagy inhibitor, namely 3‐methyladenine 70. The transplantation of vitamin E‐pretreated MSCs increases the proteoglycan content in the cartilage matrix for chondrogenic differentiation of MSCs 54. Preconditioning with all‐trans retinoic acid (ATRA) increases the expression levels of cytochrome *c* oxidase (COX)‐2, HIF‐1, CXCR4, CCR2, VEGF, angiogenin‐2 and angiogenin‐4 in MSCs, which can be reversed by a selective COX‐2 inhibitor, namely celecoxib, *in vitro*; ATRA‐pretreated MSCs enhance tube formation and the *in vivo* wound healing ability 71. Pretreatment of umbilical cord‐derived MSCs with polyribocytidylic acid enhances the therapeutic efficiency in trinitrobenzene sulphonate (TNBS)‐induced colitis mouse models; Notch‐1 signalling promotes the release of prostaglandin E2 for immune suppression of umbilical cord‐derived MSCs 72. Although drugs exert their specific protective effects on MSCs and promote the repair of injured tissues via enhancing MSC activities after transplantation, whether the drugs have adverse effects on MSC properties should be further investigated for their safe application.

In regenerative medicine, small molecules are applied to generate iPSCs from somatic cells and contribute to current cell and tissue engineering. They have been demonstrated to participate in the regulation of MSC fate *in vitro* and *in vivo*, although whether the effects are advantageous or disadvantageous should be further investigated. As shown above, mechanical stretch increases VEGFA expression and resistance to apoptosis. A small molecule, namely BAY 11‐708, blocks the pro‐angiogenesis and antiapoptosis function of MSCs via the inhibition of NF‐κB activity 73. As a naturally occurring antimicrobial peptide that can effectively enhance the proliferative and migratory abilities of MSCs to assist in wound repair, LL‐37 has been demonstrated to contribute to the improvement of MSC proliferation and migration by activation of the MAPK signalling pathway 74. Dimethyloxalylglycine (DMOG) is a prolyl hydroxylase inhibitor that induces the migration of MSCs into peripheral blood circulation. However, Ge *et al*. 75 showed that DOMG‐pretreated MSCs had a similar immune phenotype and multilineage differentiation capability as control MSCs, and the release levels of transforming growth factor (TGF) and PDGF were comparable in both groups. DMOG can effectively increase the expression levels of survival and angiogenic factors including HIF‐1α, VEGF, glucose transporter 1 and phospho‐AKT in MSCs and improve the therapeutic effects of MSCs via reducing heart infarct size and promoting functional repair in myocardial infarction after transplantation 76. Pretreatment with RU486, an antagonist of the glucocorticoid receptor, significantly increases the proliferation of human MSCs in a gender‐dependent way and enhances the expression of osteogenic markers in osteogenic MSCs compared to that observed in control MSCs 77. Moreover, preconditioning with JI‐34, a growth hormone‐releasing hormone agonist, enhances the differentiation into endothelial tube cells *in vitro* and improves the engraftment of MSCs into hemic hindlimb muscles for repairing injured parts 78. Although small molecules are widely used in current reprogramming programmes, they are still a negligible portion of active factors for preconditioning of MSCs, and a large number of small molecules should be developed for the protection of MSCs.

## Trophic factors and cytokines for regulation of MSC fate

The interaction between growth factor and its receptors activates the downstream signal transduction for cell survival and cell differentiation; thus, growth factor and cytokine preconditioning may influence the host tissue through paracrine/autocrine pathways. MSCs contain a variety of trophic factors or cytokines with biological functions including immunomodulatory properties 79 and anti‐inflammatory capacities 80. Despite the promising results, one impediment is that the short biological half‐lives of the growth factors inhibit the stable therapeutic effects. SDF‐1 pretreatment protects MSCs from H_2_O_2_‐induced apoptosis via enhancing the proliferation, migration and survival rate; concurrently, angiogenic cytokines including BFGF and VEGF and pro‐survival signalling pathways including AKT and ERK are also up‐regulated by preconditioning with SDF‐1 81. Intriguingly, TGF‐β1 drives MSC fate towards osteoblasts generation *in vitro*, while it inhibits adipogenic differentiation; furthermore, the addition of TGF‐β1 into adipogenic medium switches MSCs from adipogenesis into osteogenesis, mainly through activating SMAD/C/EBPs/PPARγ signalling pathways 82. A short‐term preconditioning with TNF‐α on MSCs enhances proliferation, mobilization and osteogenic differentiation via activation of ERK1/2 and MAPK signalling pathways, whereas silencing by siRNA can partially inhibit ERK1/2 signalling and osteogenic differentiation of MSCs 83. Pretreatment of MSCs with interferon (IFN)‐γ suppresses natural killer (NK) activation and NK‐mediated cytotoxicity partly via up‐regulating the synthesis of indoleamine 2,3‐dioxygenase (IDO) and prostaglandin E2 84. On the other hand, an optimal combination of growth factors for directed differentiation of stem cells into the desired lineage remains a challenge. IL‐1 and TNF‐α inhibit the osteogenesis and adipocyte generation of MSCs via activating the canonical NF‐κB signalling 85 and IL‐1R1/MyD88 signalling pathway 86. Moreover, the addition of TNF‐α and IFN‐γ into MSCs *in vitro* increases the production of IDO for the generation of anti‐inflammatory M2 macrophages and the suppression of human peripheral blood mononuclear cell proliferation 87. A sequential pretreatment with BFGF followed by sex steroid hormones highly improves the expression of neural markers and promotes the differentiation efficacy of bone marrow‐derived MSCs 88.

Preconditioning with various cytokines have also been proven to have protective effects on injury models and can participate in the regulation of cell fate. Preconditioning with IL‐1β increases the expression levels of various cytokines including TNF‐α, IL‐6, IL‐8 and IL‐23A and chemokines such as CCL5, CCL20, CXCL1, CXCL3, CXCL5, CXCL6, CXCL10 and CXCL11, as well as adhesion molecules such as vascular cell adhesion molecule (VCAM)‐1, intercellular adhesion molecule (ICAM)‐1 and ICAM‐4 in MSCs, thus improving the migration ability of MSCs to the site of inflammation *in vivo* 89. In addition, IL‐1β pretreatment not only increases the proliferation but also up‐regulates the chondrogenic potential of synovial MSCs; however, high concentrations of IL‐1β exert adverse effects on synovial MSCs by reducing the adhesion ability and pluripotency 90. TGF‐β1, which can be released from the bone matrix, induces MSC migration into the remodelling sites and couples bone formation and resorption via the canonical SMADs signalling pathway or the non‐canonical signalling pathways involving AKT, ERK1/2, FAK and p38 91. A low concentration of TGF‐β1 has the optimal effect on human umbilical cord‐derived MSC proliferation and stimulates the expression of ECM‐related genes without altering their immunophenotype and differentiation capacity, whereas pretreatment can significantly improve the survival of umbilical cord‐derived MSCs in damaged lungs *in vivo* 92. Preconditioning with oncostatin M (OSM), which belongs to the IL‐6 family, significantly increases the expression of type 2 OSM receptor and HGF in MSCs, consequently maintaining pulmonary respiratory function and down‐regulating the release of inflammatory and fibrotic factors in bleomycin‐induced lung fibrotic mice 93. IFN‐α‐pretreated MSCs can be used to treat dextran sodium sulphate (DSS)‐ and TNBS‐induced colitis via increasing the migration potential of MSCs and inhibiting Th1 inflammatory responses 94. Even under conditions of H/SD, pretreatment with migration inhibitory factor (MIF) significantly reduces the apoptosis rate of aged MSCs and rejuvenates the senescence by activating the AMPK‐FOXO3a signalling pathway 95. These trophic factors and growth factors may influence MSC properties synergistically or antagonistically; thus, the concentration or combination of these factors needs to be optimized according to their chemical characteristics.

## Preconditioning with physical factors and materials for regulating MSC fate

Physical factors are generally noxious for MSC activities *in vitro*; however, an increasing number of studies have proven that they could enhance the proliferation and differentiation abilities *in vitro* and *in vivo*. Extremely low‐level lasers are effective for improving the proliferation rate via increasing the S‐phase proportion and enhancing mitochondrial biogenesis via up‐regulating the generation of ROS and NO in MSCs; moreover, the migration ability of MSCs is improved by activating the ERK1/2 and FAK signalling pathways and up‐regulating expression levels of HGF and platelet‐derived growth factor (PDGF) 96. Pretreatment with pulsed electromagnetic fields (PEMF) increases the expression levels of MSCs and decreases cell death by up‐regulating the expression of AKT and RAS in a dose‐ and time‐dependent manner 97. After suspension in culture medium, silica can be divided into NPs and MPs according to its differently sized particles, and NPs increase the proliferation of adipose‐derived MSCs via promoting phosphorylation of ERK1/2, while MPs cause a slight increase in apoptosis via increasing phosphorylation of p38 98. Intriguingly, some external elements only exert their protective effects under harmful microenvironments. For instance, mechanical stretch preconditioning increases the angiogenic capacity and survival rate of MSCs via up‐regulation of VEGFA and NF‐κB p65 activity in a condition of nutrient deprivation 73. GFc7, a nanochelating‐based nanocomplex, improves cell proliferation, expression of pluripotency genes and homing markers and antioxidative defence; spontaneous differentiation‐related genes are repressed, while specific differentiation‐related genes for the direction of the determined cell fate are significantly up‐regulated 99. Selenite recovers the osteoblastic differentiation of MSCs and inhibits the activation of the ERK signalling pathway in MSCs under the conditions of excessive H_2_O_2_ via improving the activity of glutathione peroxidase and the total antioxidant capacity and reducing glutathione for ROS generation 100. Pretreatment with carboxyl‐terminated hyperbranched polyester (CHBP) maintains the MMP and mitochondrial membrane integrity and activates the Nrf2/Sirt3/FoxO3a pathway, thus providing more resistance to starvation‐induced oxidative stress in a dose‐dependent manner, while CHBP exerts little effects on the differentiation and self‐renewal capacity of MSCs under normal conditions 101. The three‐dimensional microenvironment mimics the physical environment for MSC culture by providing intensive cell–cell interactions, providing enough space for MSC proliferation and generating more biochemical and biomechanical cues 102; thus, multiple physical factors can be applied to organization of three‐dimensional microenvironment *in vitro* or *in vivo* according to their advantages. However, the usage of physical factors and materials may effectively improve MSC‐based therapeutic effects, while it may also lead to DNA damage and apoptosis of MSCs *in vitro*.

## Conclusions

Once MSCs are isolated from original tissues and induced to proliferate *in vitro*, the surrounding environment is not sufficient for maintaining their self‐renewal and differentiation capacities indefinitely. In fact, MSCs also lose a portion of their functions after isolation and cultivation *in vitro* as the surrounding microenvironments are changed and not the same as the environment *in vivo*. In addition, after they are injected *in vivo* and migrate into damaged tissues or organs, they encounter a harsh environment coupled with death signals due to the inadequate tensegrity structure between the cells and matrix. Consequently, although cultured MSCs are widely applied in cellular transplantation, the low survival rate and high apoptosis rate lower their therapeutic effects. Various protocols including preconditioning, gene modification and various coculture methods have been tested and widely investigated in recent studies. Preconditioning can influence the biological activities of MSCs *in vitro* and *in vivo*, thus highly improving the repairing efficacy for injury and disease models. However, there are still many obstacles to determine the optimal methods for preconditioning in MSC‐based therapy. First, will the factors exert adverse effects on MSCs? Secondly, what is the applicable dose of these factors? Thirdly, will MSCs acquire tumorigenicity after pretreatment? Fourthly, how can some factors be combined to synergistically enhance MSC activities? Fifthly, more factors that can effectively protect MSCs from apoptosis should be developed or produced. Lastly, the detailed mechanisms should be further investigated as there is not a simple regulative route to protect MSCs from injury. Although MSC transplantation has been applied to multiple clinical trials, additional studies should be conducted to more precisely understand whether and how such strategies may affect the *in vivo* biological activity of transplanted cells. After these potential problems that inhibit the application of preconditioning on MSCs are solved, MSC‐based therapy may be applied more widely in current regenerative medicine and can be applied for the treatment of some terminal diseases in the near future.

## Declaration of interest

The authors declare that they have no conflict of interest.

## References

[1] Hu C , Li L . *In vitro* culture of isolated primary hepatocytes and stem cell‐derived hepatocyte‐like cells for liver regeneration. Protein Cell. 2015; 6: 562–74. 26088193 10.1007/s13238-015-0180-2 PMC4506286

[2] Thomson JA , Itskovitz‐Eldor J , Shapiro SS , *et al* Embryonic stem cell lines derived from human blastocysts. Science. 1998; 282: 1145–7. 9804556 10.1126/science.282.5391.1145

[3] Young RA . Control of the embryonic stem cell state. Cell. 2011; 144: 940–54. 21414485 10.1016/j.cell.2011.01.032 PMC3099475

[4] Takahashi K , Yamanaka S . Induction of pluripotent stem cells from mouse embryonic and adult fibroblast cultures by defined factors. Cell. 2006; 126: 663–76. 16904174 10.1016/j.cell.2006.07.024

[5] Herberts CA , Kwa MS , Hermsen HP . Risk factors in the development of stem cell therapy. J Transl Med. 2011; 9: 29. 21418664 10.1186/1479-5876-9-29 PMC3070641

[6] Salem HK , Thiemermann C . Mesenchymal stromal cells: current understanding and clinical status. Stem Cells. 2010; 28: 585–96. 19967788 10.1002/stem.269 PMC2962904

[7] Pessina A , Gribaldo L . The key role of adult stem cells: therapeutic perspectives. Curr Med Res Opin. 2006; 22: 2287–300. 17076989 10.1185/030079906X148517

[8] Ankrum JA , Ong JF , Karp JM . Mesenchymal stem cells: immune evasive, not immune privileged. Nat Biotechnol. 2014; 32: 252–60. 24561556 10.1038/nbt.2816 PMC4320647

[9] Djouad F , Bouffi C , Ghannam S , *et al* Mesenchymal stem cells: innovative therapeutic tools for rheumatic diseases. Nat Rev Rheumatol. 2009; 5: 392–9. 19568253 10.1038/nrrheum.2009.104

[10] Chen Q , Shou P , Zheng C , *et al* Fate decision of mesenchymal stem cells: adipocytes or osteoblasts? Cell Death Differ. 2016; 23: 1128–39. 26868907 10.1038/cdd.2015.168 PMC4946886

[11] Tao H , Han Z , Han ZC , *et al* Proangiogenic features of mesenchymal stem cells and their therapeutic applications. Stem Cells Int. 2016; 2016: 1314709. 26880933 10.1155/2016/1314709 PMC4736816

[12] Sandvig I , Gadjanski I , Vlaski‐Lafarge M , *et al* Strategies to enhance implantation and survival of stem cells after their injection in ischemic neural tissue. Stem Cells Dev. 2017; 26: 554–65. 28103744 10.1089/scd.2016.0268

[13] Bianco P , Cao X , Frenette PS , *et al* The meaning, the sense and the significance: translating the science of mesenchymal stem cells into medicine. Nat Med. 2013; 19: 35–42. 23296015 10.1038/nm.3028 PMC3998103

[14] Madrigal M , Rao KS , Riordan NH . A review of therapeutic effects of mesenchymal stem cell secretions and induction of secretory modification by different culture methods. J Transl Med. 2014; 12: 260. 25304688 10.1186/s12967-014-0260-8 PMC4197270

[15] Mok PL , Leong CF , Cheong SK . Cellular mechanisms of emerging applications of mesenchymal stem cells. Malays J Pathol. 2013; 35: 17–32. 23817392

[16] Webster RA , Blaber SP , Herbert BR , *et al* The role of mesenchymal stem cells in veterinary therapeutics ‐ a review. N Z Vet J. 2012; 60: 265–72. 22646715 10.1080/00480169.2012.683377

[17] Singec I , Snyder EY . Inflammation as a matchmaker: revisiting cell fusion. Nat Cell Biol. 2008; 10: 503–5. 18454127 10.1038/ncb0508-503

[18] Eschenhagen T , Bolli R , Braun T , *et al* Cardiomyocyte Regeneration: A Consensus Statement. Circulation. 2017; 136: 680–6. 28684531 10.1161/CIRCULATIONAHA.117.029343 PMC5557671

[19] Choi JR , Pingguan‐Murphy B , Wan Abas WA , *et al* In situ normoxia enhances survival and proliferation rate of human adipose tissue‐derived stromal cells without increasing the risk of tumourigenesis. PLoS ONE. 2015; 10: e0115034. 25615717 10.1371/journal.pone.0115034 PMC4304807

[20] Choi JR , Pingguan‐Murphy B , Wan Abas WA , *et al* Impact of low oxygen tension on stemness, proliferation and differentiation potential of human adipose‐derived stem cells. Biochem Biophys Res Commun. 2014; 448: 218–24. 24785372 10.1016/j.bbrc.2014.04.096

[21] Schive SW , Mirlashari MR , Hasvold G , *et al* Human adipose‐derived mesenchymal stem cells respond to short‐term hypoxia by secreting factors beneficial for human islets *in vitro* and potentiate antidiabetic effect *in vivo* . Cell Med. 2017; 9: 103–16. 28713640 10.3727/215517917X693401 PMC5509020

[22] Han KH , Kim AK , Kim MH , *et al* Enhancement of angiogenic effects by hypoxia‐preconditioned human umbilical cord‐derived mesenchymal stem cells in a mouse model of hindlimb ischemia. Cell Biol Int. 2016; 40: 27–35. 26222206 10.1002/cbin.10519

[23] Barros S , Dehez S , Arnaud E , *et al* Aging‐related decrease of human ASC angiogenic potential is reversed by hypoxia preconditioning through ROS production. Mol Ther. 2013; 21: 399–408. 23070114 10.1038/mt.2012.213 PMC3594015

[24] Song L , Yang YJ , Dong QT , *et al* Atorvastatin enhance efficacy of mesenchymal stem cells treatment for swine myocardial infarction via activation of nitric oxide synthase. PLoS One. 2013; 8: e65702. 23741509 10.1371/journal.pone.0065702 PMC3669282

[25] Robey TE , Saiget MK , Reinecke H , *et al* Systems approaches to preventing transplanted cell death in cardiac repair. J Mol Cell Cardiol. 2008; 45: 567–81. 18466917 10.1016/j.yjmcc.2008.03.009 PMC2587485

[26] Majzunova M , Dovinova I , Barancik M , *et al* Redox signaling in pathophysiology of hypertension. J Biomed Sci. 2013; 20: 69. 24047403 10.1186/1423-0127-20-69 PMC3815233

[27] Chang W , Song BW , Moon JY , *et al* Anti‐death strategies against oxidative stress in grafted mesenchymal stem cells. Histol Histopathol. 2013; 28: 1529–36. 23760682 10.14670/HH-28.1529

[28] Khansari N , Shakiba Y , Mahmoudi M . Chronic inflammation and oxidative stress as a major cause of age‐related diseases and cancer. Recent Pat Inflamm Allergy Drug Discov. 2009; 3: 73–80. 19149749 10.2174/187221309787158371

[29] Saparov A , Ogay V , Nurgozhin T , *et al* Preconditioning of human mesenchymal stem cells to enhance their regulation of the immune response. Stem Cells Int. 2016; 2016: 3924858. 27822228 10.1155/2016/3924858 PMC5086389

[30] Sen S , Domingues CC , Rouphael C , *et al* Genetic modification of human mesenchymal stem cells helps to reduce adiposity and improve glucose tolerance in an obese diabetic mouse model. Stem Cell Res Ther. 2015; 6: 242. 26652025 10.1186/s13287-015-0224-9 PMC4674936

[31] Qazi TH , Mooney DJ , Duda GN , *et al* Biomaterials that promote cell‐cell interactions enhance the paracrine function of MSCs. Biomaterials. 2017; 140: 103–14. 28644976 10.1016/j.biomaterials.2017.06.019

[32] Brahimi‐Horn MC , Pouyssegur J . Oxygen, a source of life and stress. FEBS Lett. 2007; 581: 3582–91. 17586500 10.1016/j.febslet.2007.06.018

[33] Kheirandish M , Gavgani SP , Samiee S . The effect of hypoxia preconditioning on the neural and stemness genes expression profiling in human umbilical cord blood mesenchymal stem cells. Transfus Apher Sci. 2017; 56: 392–9. 28428031 10.1016/j.transci.2017.03.015

[34] Kim YS , Noh MY , Cho KA , *et al* Hypoxia/reoxygenation‐preconditioned human bone marrow‐derived mesenchymal stromal cells rescue ischemic rat cortical neurons by enhancing trophic factor release. Mol Neurobiol. 2015; 52: 792–803. 25288154 10.1007/s12035-014-8912-5

[35] Boyette LB , Creasey OA , Guzik L , *et al* Human bone marrow‐derived mesenchymal stem cells display enhanced clonogenicity but impaired differentiation with hypoxic preconditioning. Stem Cells Transl Med. 2014; 3: 241–54. 24436440 10.5966/sctm.2013-0079 PMC3925051

[36] Liu L , Gao J , Yuan Y , *et al* Hypoxia preconditioned human adipose derived mesenchymal stem cells enhance angiogenic potential via secretion of increased VEGF and bFGF. Cell Biol Int. 2013; 37: 551–60. 23505143 10.1002/cbin.10097

[37] Bader AM , Klose K , Bieback K , *et al* Hypoxic preconditioning increases survival and pro‐angiogenic capacity of human cord blood mesenchymal stromal cells *in vitro* . PLoS One. 2015; 10: e0138477. 26380983 10.1371/journal.pone.0138477 PMC4575058

[38] Estrada JC , Albo C , Benguria A , *et al* Culture of human mesenchymal stem cells at low oxygen tension improves growth and genetic stability by activating glycolysis. Cell Death Differ. 2012; 19: 743–55. 22139129 10.1038/cdd.2011.172 PMC3321628

[39] Liu J , Hao H , Huang H , *et al* Hypoxia regulates the therapeutic potential of mesenchymal stem cells through enhanced autophagy. Int J Low Extrem Wounds. 2015; 14: 63–72. 25759412 10.1177/1534734615573660

[40] Sun J , Wei ZZ , Gu X , *et al* Intranasal delivery of hypoxia‐preconditioned bone marrow‐derived mesenchymal stem cells enhanced regenerative effects after intracerebral hemorrhagic stroke in mice. Exp Neurol. 2015; 272: 78–87. 25797577 10.1016/j.expneurol.2015.03.011

[41] Yu J , Yin S , Zhang W , *et al* Hypoxia preconditioned bone marrow mesenchymal stem cells promote liver regeneration in a rat massive hepatectomy model. Stem Cell Res Ther. 2013; 4: 83. 23856418 10.1186/scrt234 PMC3854783

[42] Chang CP , Chio CC , Cheong CU , *et al* Hypoxic preconditioning enhances the therapeutic potential of the secretome from cultured human mesenchymal stem cells in experimental traumatic brain injury. Clin Sci (Lond). 2013; 124: 165–76. 22876972 10.1042/CS20120226

[43] Wei L , Fraser JL , Lu ZY , *et al* Transplantation of hypoxia preconditioned bone marrow mesenchymal stem cells enhances angiogenesis and neurogenesis after cerebral ischemia in rats. Neurobiol Dis. 2012; 46: 635–45. 22426403 10.1016/j.nbd.2012.03.002 PMC3353023

[44] Hu X , Xu Y , Zhong Z , *et al* A large‐scale investigation of hypoxia‐preconditioned allogeneic mesenchymal stem cells for myocardial repair in nonhuman primates: paracrine activity without remuscularization. Circ Res. 2016; 118: 970–83. 26838793 10.1161/CIRCRESAHA.115.307516 PMC4894788

[45] Wang X , Liu C , Li S , *et al* Hypoxia precondition promotes adipose‐derived mesenchymal stem cells based repair of diabetic erectile dysfunction via augmenting angiogenesis and neuroprotection. PLoS One. 2015; 10: e0118951. 25790284 10.1371/journal.pone.0118951 PMC4366267

[46] Cruz FF , Rocco PR . Hypoxic preconditioning enhances mesenchymal stromal cell lung repair capacity. Stem Cell Res Ther. 2015; 6: 130. 26169784 10.1186/s13287-015-0120-3 PMC4501085

[47] Feng Y , Zhu M , Dangelmajer S , *et al* Hypoxia‐cultured human adipose‐derived mesenchymal stem cells are non‐oncogenic and have enhanced viability, motility, and tropism to brain cancer. Cell Death Dis. 2015; 6: e1797. 26111059 10.1038/cddis.2015.176 PMC4669846

[48] Lee JH , Yoon YM , Lee SH . Hypoxic preconditioning promotes the bioactivities of mesenchymal stem cells via the HIF‐1alpha‐GRP78‐Akt Axis. Int J Mol Sci. 2017; 18: pii: E1320. 10.3390/ijms18061320 PMC5486141 28635661

[49] Han YS , Lee JH , Yoon YM , *et al* Hypoxia‐induced expression of cellular prion protein improves the therapeutic potential of mesenchymal stem cells. Cell Death Dis. 2016; 7: e2395. 27711081 10.1038/cddis.2016.310 PMC5133977

[50] Fan L , Zhang C , Yu Z , *et al* Transplantation of hypoxia preconditioned bone marrow mesenchymal stem cells enhances angiogenesis and osteogenesis in rabbit femoral head osteonecrosis. Bone. 2015; 81: 544–53. 26363339 10.1016/j.bone.2015.09.005

[51] Liu J , Hao H , Xia L , *et al* Hypoxia pretreatment of bone marrow mesenchymal stem cells facilitates angiogenesis by improving the function of endothelial cells in diabetic rats with lower ischemia. PLoS One. 2015; 10: e0126715. 25996677 10.1371/journal.pone.0126715 PMC4440823

[52] Hu L , Wen Y , Xu J , *et al* Pretreatment with bisphosphonate enhances osteogenesis of bone marrow mesenchymal stem cells. Stem Cells Dev. 2017; 26: 123–32. 27736364 10.1089/scd.2016.0173

[53] Ferrer RA , Wobus M , List C , *et al* Mesenchymal stromal cells from patients with myelodysplastic syndrome display distinct functional alterations that are modulated by lenalidomide. Haematologica. 2013; 98: 1677–85. 23716561 10.3324/haematol.2013.083972 PMC3815166

[54] Bhatti FU , Mehmood A , Latief N , *et al* Vitamin E protects rat mesenchymal stem cells against hydrogen peroxide‐induced oxidative stress *in vitro* and improves their therapeutic potential in surgically‐induced rat model of osteoarthritis. Osteoarthritis Cartilage. 2017; 25: 321–31. 27693502 10.1016/j.joca.2016.09.014

[55] Wang J , Li Z , Zhang Y , *et al* CX43 change in LPS preconditioning against apoptosis of mesenchymal stem cells induced by hypoxia and serum deprivation is associated with ERK signaling pathway. Mol Cell Biochem. 2013; 380: 267–75. 23712704 10.1007/s11010-013-1683-x

[56] Sun X , Fang B , Zhao X , *et al* Preconditioning of mesenchymal stem cells by sevoflurane to improve their therapeutic potential. PLoS One. 2014; 9: e90667. 24599264 10.1371/journal.pone.0090667 PMC3944720

[57] An SY , Han J , Lim HJ , *et al* Valproic acid promotes differentiation of hepatocyte‐like cells from whole human umbilical cord‐derived mesenchymal stem cells. Tissue Cell. 2014; 46: 127–35. 24472423 10.1016/j.tice.2013.12.006

[58] Li M , Yu L , She T , *et al* Astragaloside IV attenuates Toll‐like receptor 4 expression via NF‐kappaB pathway under high glucose condition in mesenchymal stem cells. Eur J Pharmacol. 2012; 696: 203–9. 23041150 10.1016/j.ejphar.2012.09.033

[59] Lee J , Shin MS , Kim MO , *et al* Apple ethanol extract promotes proliferation of human adult stem cells, which involves the regenerative potential of stem cells. Nutr Res. 2016; 36: 925–36. 27632912 10.1016/j.nutres.2016.06.010

[60] Luo G , Xu B , Huang Y . Icariside II promotes the osteogenic differentiation of canine bone marrow mesenchymal stem cells via the PI3K/AKT/mTOR/S6K1 signaling pathways. Am J Transl Res. 2017; 9: 2077–87. 28559962 PMC5446494

[61] Zhang LY , Xue HG , Chen JY , *et al* Genistein induces adipogenic differentiation in human bone marrow mesenchymal stem cells and suppresses their osteogenic potential by upregulating PPARgamma. Exp Ther Med. 2016; 11: 1853–8. 27168816 10.3892/etm.2016.3120 PMC4840518

[62] Noiseux N , Borie M , Desnoyers A , *et al* Preconditioning of stem cells by oxytocin to improve their therapeutic potential. Endocrinology. 2012; 153: 5361–72. 23024264 10.1210/en.2012-1402

[63] Najafi R , Sharifi AM . Deferoxamine preconditioning potentiates mesenchymal stem cell homing *in vitro* and in streptozotocin‐diabetic rats. Expert Opin Biol Ther. 2013; 13: 959–72. 23536977 10.1517/14712598.2013.782390

[64] Khan I , Ali A , Akhter MA , *et al* Preconditioning of mesenchymal stem cells with 2,4‐dinitrophenol improves cardiac function in infarcted rats. Life Sci. 2016; 162: 60–9. 27543341 10.1016/j.lfs.2016.08.014

[65] Liu C , Fan Y , Zhou L , *et al* Pretreatment of mesenchymal stem cells with angiotensin II enhances paracrine effects, angiogenesis, gap junction formation and therapeutic efficacy for myocardial infarction. Int J Cardiol. 2015; 188: 22–32. 25880576 10.1016/j.ijcard.2015.03.425

[66] Numasawa Y , Kimura T , Miyoshi S , *et al* Treatment of human mesenchymal stem cells with angiotensin receptor blocker improved efficiency of cardiomyogenic transdifferentiation and improved cardiac function via angiogenesis. Stem Cells. 2011; 29: 1405–14. 21755575 10.1002/stem.691

[67] Kim YS , Kwon JS , Hong MH , *et al* Restoration of angiogenic capacity of diabetes‐insulted mesenchymal stem cells by oxytocin. BMC Cell Biol. 2013; 14: 38. 24024790 10.1186/1471-2121-14-38 PMC3827832

[68] Jeong SG , Ohn T , Kim SH , *et al* Valproic acid promotes neuronal differentiation by induction of neuroprogenitors in human bone‐marrow mesenchymal stromal cells. Neurosci Lett. 2013; 554: 22–7. 24021810 10.1016/j.neulet.2013.08.059

[69] Tang Y , Cai B , Yuan F , *et al* Melatonin pretreatment improves the survival and function of transplanted mesenchymal stem cells after focal cerebral ischemia. Cell Transplant. 2014; 23: 1279–91. 23635511 10.3727/096368913x667510

[70] Wan Y , Zhuo N , Li Y , *et al* Autophagy promotes osteogenic differentiation of human bone marrow mesenchymal stem cell derived from osteoporotic vertebrae. Biochem Biophys Res Commun. 2017; 488: 46–52. 28476617 10.1016/j.bbrc.2017.05.004

[71] Pourjafar M , Saidijam M , Mansouri K , *et al* All‐trans retinoic acid preconditioning enhances proliferation, angiogenesis and migration of mesenchymal stem cell *in vitro* and enhances wound repair *in vivo* . Cell Prolif. 2017; 50: e12315. 10.1111/cpr.12315 PMC6529123 27862498

[72] Qiu Y , Guo J , Mao R , *et al* TLR3 preconditioning enhances the therapeutic efficacy of umbilical cord mesenchymal stem cells in TNBS‐induced colitis via the TLR3‐Jagged‐1‐Notch‐1 pathway. Mucosal Immunol. 2017; 10: 727–42. 27649928 10.1038/mi.2016.78

[73] Zhu Z , Gan X , Fan H , *et al* Mechanical stretch endows mesenchymal stem cells stronger angiogenic and anti‐apoptotic capacities via NFkappaB activation. Biochem Biophys Res Commun. 2015; 468: 601–5. 26545780 10.1016/j.bbrc.2015.10.157

[74] Yang Y , Choi H , Seon M , *et al* LL‐37 stimulates the functions of adipose‐derived stromal/stem cells via early growth response 1 and the MAPK pathway. Stem Cell Res Ther. 2016; 7: 58. 27095351 10.1186/s13287-016-0313-4 PMC4837546

[75] Ge T , Yu Q , Liu W , *et al* Characterization of bone marrow‐derived mesenchymal stem cells from dimethyloxallyl glycine‐preconditioned mice: evaluation of the feasibility of dimethyloxallyl glycine as a mobilization agent. Mol Med Rep. 2016; 13: 3498–506. 26935134 10.3892/mmr.2016.4945 PMC4805059

[76] Liu XB , Wang JA , Ji XY , *et al* Preconditioning of bone marrow mesenchymal stem cells by prolyl hydroxylase inhibition enhances cell survival and angiogenesis *in vitro* and after transplantation into the ischemic heart of rats. Stem Cell Res Ther. 2014; 5: 111. 25257482 10.1186/scrt499 PMC4535299

[77] Yu Y , Wei N , Stanford C , *et al* *In vitro* effects of RU486 on proliferation and differentiation capabilities of human bone marrow mesenchymal stromal cells. Steroids. 2012; 77: 132–7. 22093480 10.1016/j.steroids.2011.10.017 PMC3242919

[78] Ma Q , Xia X , Tao Q , *et al* Profound actions of an agonist of growth hormone‐releasing hormone on angiogenic therapy by mesenchymal stem cells. Arterioscler Thromb Vasc Biol. 2016; 36: 663–72. 26868211 10.1161/ATVBAHA.116.307126 PMC4808467

[79] Bernardo ME , Fibbe WE . Mesenchymal stromal cells: sensors and switchers of inflammation. Cell Stem Cell. 2013; 13: 392–402. 24094322 10.1016/j.stem.2013.09.006

[80] Keating A . Mesenchymal stromal cells: new directions. Cell Stem Cell. 2012; 10: 709–16. 22704511 10.1016/j.stem.2012.05.015

[81] Liu X , Duan B , Cheng Z , *et al* SDF‐1/CXCR4 axis modulates bone marrow mesenchymal stem cell apoptosis, migration and cytokine secretion. Protein Cell. 2011; 2: 845–54. 22058039 10.1007/s13238-011-1097-z PMC4875294

[82] van Zoelen EJ , Duarte I , Hendriks JM , *et al* TGFbeta‐induced switch from adipogenic to osteogenic differentiation of human mesenchymal stem cells: identification of drug targets for prevention of fat cell differentiation. Stem Cell Res Ther. 2016; 7: 123. 27562730 10.1186/s13287-016-0375-3 PMC5000485

[83] Lu Z , Wang G , Dunstan CR , *et al* Activation and promotion of adipose stem cells by tumour necrosis factor‐alpha preconditioning for bone regeneration. J Cell Physiol. 2013; 228: 1737–44. 23359411 10.1002/jcp.24330

[84] Noone C , Kihm A , English K , *et al* IFN‐gamma stimulated human umbilical‐tissue‐derived cells potently suppress NK activation and resist NK‐mediated cytotoxicity *in vitro* . Stem Cells Dev. 2013; 22: 3003–14. 23795941 10.1089/scd.2013.0028 PMC3824722

[85] Sullivan CB , Porter RM , Evans CH , *et al* TNFalpha and IL‐1beta influence the differentiation and migration of murine MSCs independently of the NF‐kappaB pathway. Stem Cell Res Ther. 2014; 5: 104. 25163844 10.1186/scrt492 PMC4177434

[86] Martino MM , Maruyama K , Kuhn GA , *et al* Inhibition of IL‐1R1/MyD88 signalling promotes mesenchymal stem cell‐driven tissue regeneration. Nat Commun. 2016; 7: 11051. 27001940 10.1038/ncomms11051 PMC4804175

[87] Francois M , Romieu‐Mourez R , Li M , *et al* Human MSC suppression correlates with cytokine induction of indoleamine 2,3‐dioxygenase and bystander M2 macrophage differentiation. Mol Ther. 2012; 20: 187–95. 21934657 10.1038/mt.2011.189 PMC13064680

[88] Parivar K , Baharara J , Sheikholeslami A . Neural differentiation of mouse bone marrow‐derived mesenchymal stem cells treated with sex steroid hormones and basic fibroblast growth factor. Cell J. 2015; 17: 27–36. 25870832 10.22074/cellj.2015.509 PMC4393669

[89] Carrero R , Cerrada I , Lledo E , *et al* IL1beta induces mesenchymal stem cells migration and leucocyte chemotaxis through NF‐kappaB. Stem Cell Rev. 2012; 8: 905–16. 22467443 10.1007/s12015-012-9364-9 PMC3412085

[90] Matsumura E , Tsuji K , Komori K , *et al* Pretreatment with IL‐1beta enhances proliferation and chondrogenic potential of synovium‐derived mesenchymal stem cells. Cytotherapy. 2017; 19: 181–93. 27979606 10.1016/j.jcyt.2016.11.004

[91] Dubon MJ , Yu J , Choi S , *et al* Transforming growth factor beta induces bone marrow mesenchymal stem cell migration via noncanonical signals and N‐cadherin. J Cell Physiol. 2017; 233: 201–13. 28213973 10.1002/jcp.25863

[92] Li D , Liu Q , Qi L , *et al* Low levels of TGF‐beta1 enhance human umbilical cord‐derived mesenchymal stem cell fibronectin production and extend survival time in a rat model of lipopolysaccharide‐induced acute lung injury. Mol Med Rep. 2016; 14: 1681–92. 27357811 10.3892/mmr.2016.5416

[93] Lan YW , Theng SM , Huang TT , *et al* Oncostatin M‐preconditioned mesenchymal stem cells alleviate bleomycin‐induced pulmonary fibrosis through paracrine effects of the hepatocyte growth factor. Stem Cells Transl Med. 2017; 6: 1006–17. 28297588 10.5966/sctm.2016-0054 PMC5442768

[94] Duijvestein M , Wildenberg ME , Welling MM , *et al* Pretreatment with interferon‐gamma enhances the therapeutic activity of mesenchymal stromal cells in animal models of colitis. Stem Cells. 2011; 29: 1549–58. 21898680 10.1002/stem.698

[95] Xia W , Zhang F , Xie C , *et al* Macrophage migration inhibitory factor confers resistance to senescence through CD74‐dependent AMPK‐FOXO3a signaling in mesenchymal stem cells. Stem Cell Res Ther. 2015; 6: 82. 25896286 10.1186/s13287-015-0076-3 PMC4453287

[96] Yin K , Zhu R , Wang S , *et al* Low‐level laser effect on proliferation, migration, and antiapoptosis of mesenchymal stem cells. Stem Cells Dev. 2017; 26: 762–75. 28178868 10.1089/scd.2016.0332

[97] Urnukhsaikhan E , Cho H , Mishig‐Ochir T , *et al* Pulsed electromagnetic fields promote survival and neuronal differentiation of human BM‐MSCs. Life Sci. 2016; 151: 130–8. 26898125 10.1016/j.lfs.2016.02.066

[98] Kim KJ , Joe YA , Kim MK , *et al* Silica nanoparticles increase human adipose tissue‐derived stem cell proliferation through ERK1/2 activation. Int J Nanomedicine. 2015; 10: 2261–72. 25848249 10.2147/IJN.S71925 PMC4378289

[99] Hafizi M , Hajarizadeh A , Atashi A , *et al* Nanochelating based nanocomplex, GFc7, improves quality and quantity of human mesenchymal stem cells during *in vitro* expansion. Stem Cell Res Ther. 2015; 6: 226. 26597909 10.1186/s13287-015-0216-9 PMC4657224

[100] Liu H , Bian W , Liu S , *et al* Selenium protects bone marrow stromal cells against hydrogen peroxide‐induced inhibition of osteoblastic differentiation by suppressing oxidative stress and ERK signaling pathway. Biol Trace Elem Res. 2012; 150: 441–50. 22890880 10.1007/s12011-012-9488-4

[101] Wang S , Zhang C , Niyazi S , *et al* A novel cytoprotective peptide protects mesenchymal stem cells against mitochondrial dysfunction and apoptosis induced by starvation via Nrf2/Sirt3/FoxO3a pathway. J Transl Med. 2017; 15: 33. 28202079 10.1186/s12967-017-1144-5 PMC5309997

[102] Sart S , Agathos SN , Li Y , *et al* Regulation of mesenchymal stem cell 3D microenvironment: from macro to microfluidic bioreactors. Biotechnol J. 2016; 11: 43–57. 26696441 10.1002/biot.201500191

